# ICTV Virus Taxonomy Profile: *Rhabdoviridae*

**DOI:** 10.1099/jgv.0.001020

**Published:** 2018-02-19

**Authors:** Peter J. Walker, Kim R. Blasdell, Charles H. Calisher, Ralf G. Dietzgen, Hideki Kondo, Gael Kurath, Ben Longdon, David M. Stone, Robert B. Tesh, Noël Tordo, Nikos Vasilakis, Anna E. Whitfield, ICTV Report Consortium

**Affiliations:** ^1^​ School of Biological Sciences, University of Queensland, St. Lucia, QLD 4072, Australia; ^2^​ CSIRO Health and Biosecurity, Geelong, VIC 3220, Australia; ^3^​ Colorado State University, Fort Collins, CO 80523, USA; ^4^​ Queensland Alliance for Agriculture and Food Innovation, University of Queensland, St. Lucia, QLD 4072, Australia; ^5^​ Institute of Plant Science and Resources, Okayama University, Kurashiki, 710-0046, Japan; ^6^​ Western Fisheries Research Center, Seattle, WA 98115, USA; ^7^​ Department of Biosciences, University of Exeter, Penryn TR10 9FE, UK; ^8^​ Centre for Environment, Fisheries and Aquaculture Science, Weymouth, DT4 8UB, UK; ^9^​ Department of Pathology and Center for Biodefense and Emerging Infectious Diseases, University of Texas Medical Branch, Galveston, TX 77555, USA; ^10^​ Institut Pasteur de Guinée, Gamal Abdel Nasser University, Conakry, Guinea; ^11^​ Department of Plant Pathology, Kansas State University, Manhattan KS 66506, USA

**Keywords:** *Rhabdoviridae*, rhabdoviruses, ICTV, Taxonomy

## Abstract

The family *Rhabdoviridae* comprises viruses with negative-sense (–) single-stranded RNA genomes of 10.8–16.1 kb. Virions are typically enveloped with bullet-shaped or bacilliform morphology but can also be non-enveloped filaments. Rhabdoviruses infect plants and animals including mammals, birds, reptiles and fish, as well as arthropods which serve as single hosts or act as biological vectors for transmission to animals or plants. Rhabdoviruses include important pathogens of humans, livestock, fish and agricultural crops. This is a summary of the International Committee on Taxonomy of Viruses (ICTV) Report on the taxonomy of *Rhabdoviridae,* which is available at www.ictv.global/report/rhabdoviridae.

## Abbreviations

N, nucleocapsid protein; L, large polymerase protein; P, phosphoprotein; M, matrix protein; G, glycoprotein; RdRP, RNA-dependent RNA polymerase; RNP, ribonucleoprotein.

## Virion

**Fig. 1.**
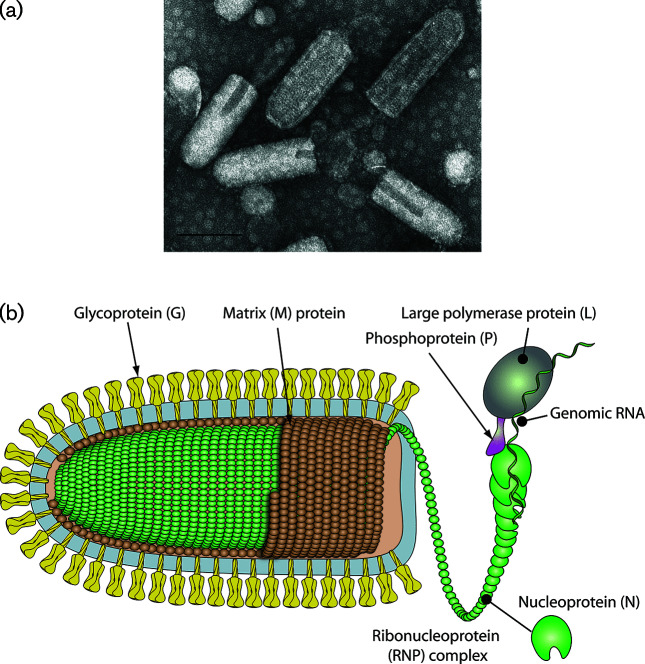
(a) Negative-contrast electron micrograph of vesicular stomatitis Indiana virus particles. The bar represents 100 nm (courtesy of P. Perrin). (b) Schematic illustration of a rhabdovirus virion and ribonucleocapsid structure. Unravelling of the RNP is illustrative to show its association with L and P (courtesy of P. Le Mercier).

Virions are usually enveloped and bullet-shaped or bacilliform (i.e. with two rounded ends) and contain five structural proteins (Table 1, Fig. 1). The nucleocapsid protein (N), the large multi-functional RNA-dependent RNA polymerase (L) and the polymerase-associated phosphoprotein (P) together with the RNA genome form the ribonucleoprotein (RNP) complex. The nucleocapsid is encased in the matrix protein (M) layer which also interacts with the envelope containing the transmembrane glycoprotein (G). Plant rhabdoviruses assigned to the genus *Varicosavirus* are filamentous and lack an envelope.

**Table 1.** Characteristics of the family *Rhabdoviridae*

**Typical member:**	vesicular stomatitis Indiana virus (AF473864), species *Indiana* v*esiculovirus*, genus *Vesiculovirus*
Virion	Bullet-shaped or bacilliform particle 100–430 nm in length and 45–100 nm in diameter comprised of a nucleocapsid surrounded by a matrix layer and a lipid envelope. Some rhabdoviruses have non-enveloped filamentous virions
Genome	Negative-sense, single-stranded RNA of 10.8–16.1 kb (unsegmented or bi-segmented)
Replication	Ribonucleoprotein (RNP) complexes containing anti-genomic RNA are generated and serve as templates for synthesis of nascent RNP complexes containing genomic RNA
Translation	Capped and polyadenylated mRNAs transcribed processively from each gene (3′ to 5′), sometimes containing multiple ORFs
Host range	Vertebrates, arthropods and plants; many vertebrate and plant rhabdoviruses are arthropod-borne
Taxonomy	18 genera containing >130 species. Many rhabdoviruses remain unclassified

## Genome

**Fig. 2.**
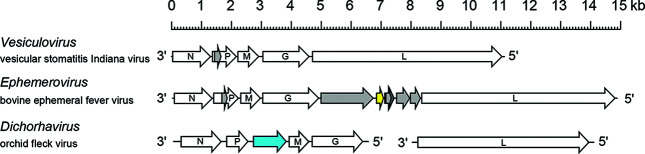
Schematic representation of rhabdovirus genome organization, exemplifying variations in architecture and the number and location of accessory genes. Arrows indicate the position of long ORFs. Alternative ORFs occur within some genes; only ORFs (≥180 nt) that appear likely to be expressed are shown.​ ORFs encoding viroporin (yellow) and ​movement proteins (blue) are shown.

Rhabdovirus negative sense (–) single-stranded RNA genomes range from 10.8 to 16.1 kb [1]. Almost all rhabdovirus genomes are unsegmented but rhabdoviruses with bi-segmented genomes are also known [2]. Terminal non-coding regions are partially complementary. Genomes usually encode five major structural proteins but may also encode additional (accessory) proteins either in additional genes or as alternative ORFs within the structural protein genes (Fig. 2) [1, 3].

## Replication

Rhabdovirus replication generally occurs in the cytoplasm following receptor-mediated endocytosis. Primary transcription is initiated from the incoming (–)RNP complex by the RNA-dependent RNA polymerase (RdRP). Stop–start transcription occurs 3′ to 5′ using gene start and gene end sequences, separated by non-transcribed intergenic sequences, to generate capped and polyadenylated mRNAs. Replication is initiated by the RdRP from a single promoter at the 3' end, ignoring gene start and end sequences to generate a (+)RNP. This is the template to generate nascent (–)RNPs which are assembled with M and G into enveloped virions. Budding can occur at either the plasma membrane or internal membranes. Some plant rhabdoviruses replicate in the nucleus.

## Taxonomy

The *Rhabdoviridae* includes 18 genera and one unassigned species (*Moussa virus*). Viruses assigned to each genus form a monophyletic clade based on phylogenetic analyses of L protein sequences and usually have similar genome organizations, including the number and locations of accessory genes. Rhabdoviruses have been isolated from a wide range of vertebrates and plants; many have been isolated from arthropods [4, 5]. Members of the genus *Lyssavirus* infect only mammals, including humans in which they can cause fatal encephalitis (rabies). Members of the genera *Vesiculovirus*, *Ephemerovirus*, *Tibrovirus*, *Hapavirus*, *Curiovirus*, *Sripuvirus* and *Ledantevirus* infect vertebrates (mammals, birds or reptiles) and are transmitted by arthropods. Some arthropod-borne rhabdoviruses are associated with diseases of livestock; some may cause disease in humans. Members of the genus *Tupavirus* have only been isolated from vertebrates. Members of the genera *Novirhabdovirus*, *Sprivivirus* and *Perhabdovirus* infect only fish, some causing economically important diseases. Rhabdoviruses assigned to the genus *Sigmavirus* each infect only dipteran flies of a single species and they are transmitted vertically. Members of the genus *Almendravirus* replicate only in insects. Plant rhabdoviruses are assigned to the genera *Cytorhabdovirus*, *Nucleorhabdovirus*, *Dichorhavirus* and *Varicosavirus* and are transmitted by either arthropods or chytrid fungi. Many are associated with diseases of agricultural or horticultural importance.

## Resources

Full ICTV Online (10th) Report: www.ictv.global/report/rhabdoviridae.
